# Rhabdomyolysis and the Use of Low-Dose Amphetamine

**DOI:** 10.7759/cureus.27357

**Published:** 2022-07-27

**Authors:** Austin R Swisher, Richard Pham, Bassam Theodory, Shawn Valiani, Nandini Gowda

**Affiliations:** 1 Department of Internal Medicine, University of California Riverside School of Medicine, Riverside, USA; 2 Department of Internal Medicine, California University of Science and Medicine, Colton, USA

**Keywords:** adhd, exercise, amphetamine, dextroamphetamine, rhabdomyolysis

## Abstract

Rhabdomyolysis ranges from being asymptomatic with elevated creatine kinase (CK) to a potentially life-threatening condition involving multiple organ systems. Muscular trauma is the most common cause, followed by enzyme deficiencies, electrolyte abnormalities, drugs, toxins, and endocrinopathies. While these risk factors are delineated, it is not clear if mild exposure to a combination of risk factors could lead to the development of rhabdomyolysis. In this case report, a 22-year-old male of Pakistani/Caucasian ethnicity presented to the emergency room with myalgias and tea-colored urine after starting a new exercise program. His serum CK level and liver function tests were significantly elevated. He was successfully treated for acute rhabdomyolysis with aggressive hydration. However, the etiology of his condition was not clear given that his exercise was not considered vigorous. The only plausible explanation for his symptoms included the use of prescribed dextroamphetamine, which may have exacerbated the physiologic responses induced by exercise. This report describes a novel case in which a patient may have developed recurrent episodes of rhabdomyolysis due to low-dose dextroamphetamine use. The combination of exercise and dextroamphetamine use may predispose patients to develop rhabdomyolysis.

## Introduction

This article was presented as an abstract at the 2022 European Congress of Internal Medicine in Malaga, Spain.

Rhabdomyolysis is caused by the breakdown and necrosis of skeletal muscle fibers and the release of intracellular contents into the systemic circulation [1]. Clinical presentation may vary, but the release of electrolytes, myoglobin, lactate dehydrogenase, and creatine kinase (CK) can lead to potentially life-threatening conditions. These include volume depletion, metabolic abnormalities, and, in the later stages of the syndrome, acute kidney injury (AKI). Patients commonly visit the emergency room with symptoms of muscle weakness, myalgia, edema, and gross pigmentation without hematuria. In the setting of rhabdomyolysis, these symptoms are usually elicited by crush injuries, overexertion, dehydration, hypophosphatemia, or alcohol abuse. Additionally, the use of amphetamine-based compounds and stimulants such as cocaine have been identified as promoters of muscle tissue breakdown [2-5].

Attention deficit hyperactivity disorder (ADHD) is a multifaceted neuropsychiatric condition that affects approximately 3% of adults globally [6]. The rate of diagnosis in the US is as high as 11% for children between the ages of 4-17, with 6.1% of children receiving medication for ADHD [7]. Dextroamphetamine is routinely used in the treatment of ADHD for both children and adults. While clinical trials have demonstrated short-term side effects, little research exists regarding the adverse drug reactions from long-term use of dextroamphetamine [8]. Common side effects of amphetamine-based medications include dry mouth, irritability, dysphoria, reduced appetite, weight loss, headache, and constipation. Rare side effects may involve psychosis, cardiac events, sexual dysfunction, visual disturbance, dermatologic changes, shortness of breath, and the potential for misuse or addiction [9]. The use of amphetamine-based compounds at high concentrations is a well-documented risk factor for rhabdomyolysis [10,11]. However, few studies have reported rhabdomyolysis in patients using amphetamines at low, pharmacologic doses [5,12,13].

We report a case in which a patient developed rhabdomyolysis due to nontoxic concentrations of dextroamphetamine use with non-vigorous exercise as the only plausible risk factor eliciting recurrent episodes.

## Case presentation

A 22-year-old male with a history of asthma and ADHD presented to the emergency department with myalgia and dysuria after reinitiating an exercise program. The patient described a similar episode two months prior, for which he was admitted with a CK of 8,846 U/L. On this occasion, however, the patient’s admission CK was elevated at 10,335 U/L. There were no signs of renal failure and his urinalysis was normal despite a reported dark tea-color. He was subsequently admitted to the hospital for intravenous hydration and overnight observation with suspected rhabdomyolysis.

Before the pandemic forced gyms to close, the patient regularly performed cardiovascular and strength-based training. Due to the fear of provoking recurrent symptoms, the patient waited before attempting to exercise again. Two months after the first episode, he made efforts to ensure adequate hydration and then completed a 1-mile jog and 10 lb. bicep curls at low intensity. However, 3-4 days later he started experiencing extreme myalgias.

The patient denied any recent viral illness or travel, nor did he report the use of alcohol or any supplements before exercising. With the exception of marijuana, his toxicology screen was negative. He had recently switched from 10mg dextroamphetamine-amphetamine (Adderall) extended-release (XR) to 5mg immediate-release (IR) preparation.

Admission laboratory values also showed signs of transaminitis. Aminotransferases were elevated (aspartate aminotransferase (AST)>alanine aminotransferase (ALT)) with AST at 856 U/L and ALT at 285 U/L. As shown in Table 1, there was mild hyperbilirubinemia and a normal anion gap. Within hours of aggressive fluid resuscitation, CK decreased to 6,314 U/L, AST and ALT improved to 133 U/L and 67 U/L, respectively, and total bilirubin normalized to 1.2 mg/dL. His metabolic values and vital signs remained stable throughout the night without any signs of AKI. The patient’s symptoms ultimately dissipated and he was discharged within 24 hours.

**Table 1:** Laboratory values H: High; L: Low; BUN: Blood urea nitrogen; GFR: Glomerular filtration rate; AST: aspartate aminotransferase; ALT: alanine aminotransferase; CK-MB: Creatine kinase-MB; PT: Prothrombin time; INR: International normalised ratio; WBC: White blood cells; RBC: Red blood cells; Hgb: hemoglobin; Hct: Hematocrit; MCV: Mean corpuscular volume; MCH: Mean corpuscular hemoglobin; MCHC: Mean corpuscular hemoglobin concentration; RDW: Red cell distribution width; MPV: Mean platelet volume.

Chemistry	
Sodium (136 – 145 mmol/L)	137
Potassium (3.5 – 5.1 mmol/L)	4.1
Chloride (98 – 107 mmol/L)	105
Carbon Dioxide (21 – 32 mmol/L)	27
Anion Gap (5 – 15 mmol/L)	5
BUN (7 – 18 mg/dL)	10
Creatinine (0.700 – 1.30 mg/dL)	0.900
Estimated GFR	106
BUN/Creatinine Ratio (8.0 – 20.0)	11.1
Glucose (74 – 106 mg/dL)	90
Calculated Osmolality (275 – 295 mOsm/kg)	272 (L)
Uric Acid (3.5 – 7.2 mg/dL)	6.5
Calcium (8.5 – 10.1 mg/dL)	10.7 (H)
Phosphorus (2.5 – 4.9 mg/dL)	3.4
Magnesium (1.8 – 2.4 mg/dL)	2.0
Total Bilirubin (0.2 – 1.0 mg/dL)	1.5 (H)
AST (15 – 37 U/L)	856 (H)
ALT (12 – 78 U/L)	285 (H)
Total Alk Phosphatase (45 – 117 U/L)	92
Creatine Kinase (935 – 232 U/L)	10335 (H)
CK-MB (CK-2) (0.5 – 3.6 ng/mL)	10.9 (H)
Serum Total Protein (6.4 – 8.2 g/dL)	8.3 (H)
Albumin (3.4 – 5.0 g/dL) Globulin (2.5 – 4.2 g/dL)	4.7 3.6
Album/Globulin Ratio (1.0 – 2.5)	1.3
Spec Hemolysis Index	1
Coagulation	
PT (9.2 – 13.2 sec)	12.2
INR	1.0
Hematology	
WBC (4.8 – 10.8 K/mm^3^)	7.5
RBC (4.6 – 6.2 M/mm^3^)	5.02
Hgb (13.0 – 18.0 gm/dL)	15.6
Hct (38 – 54 %)	44.9
MCV (80 – 99 fL)	89.4
MCH (27 – 34 pg)	31.0
MCHC (32 – 36.9 g/dL)	34.7
RDW (11.0 – 14.5 %)	12.9
Plt Count (150 – 400 K/mm^3^)	265
MPV (7.4 – 10.4 fL)	8.7
Neutrophils % (42.2 – 75.2 %)	58.2
Lymphocytes % (21 – 51 %)	31.7
Monocytes % (0 – 15 %)	8.9
Eosinophils % (0 – 7 %)	0.5
Basophils % (0 – 2 %)	0.7
Nucleated RBCs (0 / 100 WBC)	0

To prevent potential recurrence, the patient trialed bupropion (Wellbutrin) but due to ineffective clinical response, he was switched back to Adderall XR with close monitoring of CK levels. His CK at follow-up was 123 U/L without symptoms suggestive of rhabdomyolysis.

## Discussion

Rhabdomyolysis is a condition in which damaged myocytes release intracellular contents, leading to widespread necrosis [1]. While there are numerous etiologies of rhabdomyolysis, exertional rhabdomyolysis (ER) is a rare subtype with an incidence of approximately 29.9 per 100,000 patient years [14]. ER is prompted by any type of strenuous physical activity that damages myocytes, resulting in the rapid release of ions and proteins such as myoglobin, CK, or lactate dehydrogenase. The subsequent metabolic shift and protein efflux can overwhelm the renal clearance system and precipitate AKI. A common clinical sign of this is dark-colored urine, as seen in the patient in this case.

A dramatic increase in strenuous activity in an unaccustomed individual is the primary risk factor for ER [15]. Similar to the patient, ER often occurs in individuals starting a new exercise program. Other risk factors include hydration and potassium levels, male sex, extreme temperature, and genetic factors such as sickle trait, carnitine palmitoyl transferase deficiency, and McArdle disease [16]. Given that the patient’s only additional risk factors were his sex, we suspect that amphetamine use must also be implicated in the etiology of his recurrent episodes of rhabdomyolysis.

Approximately 81% of cases of rhabdomyolysis are related to drugs, alcohol, or substance use [2]. Amphetamine-induced rhabdomyolysis is believed to arise from high-dose administration or a toxic overdose that increases monoamine concentrations within the central and peripheral nervous system. The resultant neuromuscular hyperactivity and autonomic stimulation significantly deplete myocyte energy stores. The appetite-suppressing effects of amphetamines blunt eating and drinking behavior, which further decreases adenosine triphosphate (ATP) production and volume status. Amphetamines also increase serum norepinephrine and dopamine, increasing ATP demand [10]. Thus, the compounding effects of these physiologic changes culminate with intracellular protease activation, mitochondrial damage, and systemic cell death. However, it is not clear whether low-dose administration of amphetamines (i.e. pharmacotherapy for ADHD) can produce a response of sufficient magnitude to trigger rhabdomyolysis.

Adderall and Adderall XR are a mixture of d-amphetamine and l-amphetamine salts in the ratio of 3:1, otherwise known as dextroamphetamine-amphetamine or simply dextroamphetamine. A single dose of Adderall XR 10mg gives drug levels of d-amphetamine and l-amphetamine comparable to Adderall IR 10mg administered in two divided doses four hours apart. In adults, the half-life is 10 hours for d-amphetamine and 13 hours for l-amphetamine [17]. Given these pharmacokinetics, the US Food and Drug Administration issued a warning on the medication guide for dextroamphetamine stating that it “rarely” causes rhabdomyolysis.

In this report, we present the case of an otherwise healthy patient diagnosed with rhabdomyolysis due to an unknown cause. The etiology of the rhabdomyolysis was unclear given that he did not start a very intensive exercise program, but his dextroamphetamine use for ADHD was recently changed from XR to IR preparation. This could be the underlying factor increasing susceptibility to rhabdomyolysis with more strenuous exercise. Thus, this case may reflect superimposed etiologies deriving from both the exertional and amphetamine-induced subtypes of rhabdomyolysis.

To our knowledge, only three other cases of rhabdomyolysis have been reported at nontoxic concentrations. In all three, the patients had elevated CK levels and a clinical picture fitting that of rhabdomyolysis. Dehoney and Wellein [12], as well as Sultana and Byrne [13], discuss low-dose dextroamphetamine use and heavy exercise as predisposing risk factors in their cases. Santoro et al. report exercise, dehydration, potassium depletion, and alcohol consumption as predisposing risk factors that create a 'tipping point' for symptomatic rhabdomyolysis when combined with low-dose dextroamphetamine use [5]. Our case supports these findings, although with perhaps even fewer risk factors given the patient’s history of non-vigorous exercise and no signs of dehydration or potassium depletion. Nevertheless, the combination of amphetamine use and exercise may explain the patient’s recurrent episodes of rhabdomyolysis.

The patient has been receiving regular follow-up care at an outpatient clinic. Despite the apparent stabilization of CK levels and remission of rhabdomyolysis-like symptoms, he ultimately decided to terminate treatment altogether. Several months after discontinuing Adderall, the patient denied any further medical issues and performs high-intensity exercise without restriction.

## Conclusions

As the rate of prescription and non-prescription use of amphetamine-based medications continues to rise, the possible association of rhabdomyolysis with the multiple risk factor model discussed here warrants concern and should be explored in future studies to identify and potentially screen patients who might be at risk.
